# An Assessment of Allelic and SNP Bias in a Genome‐Wide SNP Enrichment Kit for Killer Whales

**DOI:** 10.1002/ece3.73887

**Published:** 2026-07-03

**Authors:** C. D. Baumgartner, O. A. Filatova, A. D. Foote, E. Jourdain, F. I. P. Samarra, I. M. Reeves

**Affiliations:** ^1^ Department of Environmental Systems Science Swiss Federal Institute of Technology Zurich Zurich Switzerland; ^2^ Orcestra Zurich Switzerland; ^3^ Department of Biology University of Southern Denmark Odense Denmark; ^4^ Centre for Ecological and Evolutionary Synthesis, Department of Biosciences University of Oslo Oslo Norway; ^5^ Norwegian Orca Survey Andenes Norway; ^6^ Section for Aquatic Biology and Toxicology, Department of Biosciences University of Oslo Oslo Norway; ^7^ Westman Islands Research Centre, University of Iceland Vestmannaeyjar Iceland; ^8^ Cetacean Research Centre (CETREC WA) Esperance Western Australia Australia; ^9^ Southern Shark Ecology Group, College of Science and Engineering Flinders University Adelaide South Australia Australia

## Abstract

Genetic studies of killer whales ( *Orcinus orca* ) historically relied on markers from mitochondrial DNA or microsatellites, which are cost‐effective, but the difficulties in cross‐laboratory standardization have limited global data integration. Transitioning to whole genome sequencing (WGS) has advanced our understanding of killer whale population structure and demographic histories; however, the costs of WGS remain a limiting factor for studying many individuals. Targeted Single Nucleotide Polymorphism (SNP) genotyping provides a cost‐effective, reproducible alternative, yet it can be susceptible to ascertainment bias whereby markers discovered in a limited set of reference populations may fail to capture genetic diversity in unrepresented populations. Here, we evaluate ascertainment bias in a custom RNA bait enrichment kit targeting 1346 SNPs, originally identified in a dataset of North Pacific, Icelandic, and Southern Ocean killer whales. Specifically, we compare the relationships among 25 killer whales representing the species' global range inferred from 225,281 unlinked genome‐wide SNPs to the same relationships inferred from the 1346 SNPs targeted by the capture baits, with both sets subsampled from WGS data. Comparing principal component analyses (PCAs), the targeted subset recapitulated broad‐scale global population structure and clustering patterns consistent with the larger WGS dataset. We found a high correlation between the targeted bait PCA and the WGS PCA (*r* = 0.961). However, the observed value fell below expectations based on random sampling (*p* < 0.01). This deviation was likely driven by samples belonging to lineages not included in the original SNP discovery panel, suggesting a small level of ascertainment bias. Consequently, some unique genetic variation in these excluded lineages remains unrepresented, specifically impacting inference of the relationships to and among lower‐latitude populations. While this targeted enrichment panel is a robust tool for elucidating major evolutionary differentiation and facilitating cross‐laboratory comparisons, it is less reliable for detecting fine‐scale structure in unrepresented, genetically divergent populations.

## Graphical abstract

This study evaluates ascertainment bias in a targeted SNP genotyping panel for killer whales ( *Orcinus orca* ) by comparing its performance against comprehensive whole genome sequencing data. Results indicate that while the targeted panel accurately recapitulates broad‐scale global population structure, it demonstrates lower fine‐scale resolution for lineages excluded from the original marker discovery, particularly in lower‐latitude populations. Consequently, while the panel is a robust tool for major evolutionary differentiation, it may be less reliable for detecting diversity in unrepresented, genetically divergent populations.

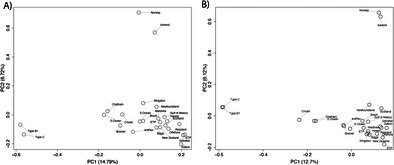

## 1 Introduction

Killer whales ( *Orcinus orca* ) occur all over the world, but regional populations can be highly specialized to local ecological conditions and have limited genetic exchange with adjacent or sympatric populations (Barrett‐Lennard 2000; Hoelzel et al. 1998, 2007; Foote et al. 2009, Stevens et al. 1989). The markers used in population genetic analyses have evolved over the past four decades, offering increasing resolution into the complex social structures and population dynamics of killer whales. Genetic studies of killer whales started from the work of Stevens et al. (1989) who analyzed fragment length polymorphism following treatment with a restriction digestion enzyme and found significant variation that allowed to distinguish North Atlantic killer whales from two North Pacific ecotypes (“resident” and “transient”). Hoelzel and Dover (1991) sequenced the D‐loop region of mitochondrial DNA showing that the North Pacific ecotypes were as genetically distinct from each other as each of them were from a South Atlantic population. Hoelzel et al. (1998) were the first to publish analyses of genotypes of killer whales inferred from microsatellite markers, and, together with Barrett‐Lennard (2000), demonstrated reproductive isolation between North Pacific ecotypes. However, a major drawback of microsatellites is that comparison among datasets generated by different laboratories can be challenging, unless several replicates are shared across laboratories to calibrate the scoring of alleles (i.e., ensuring consistent size for the same marker). Thus, population genetic studies were largely self‐contained until the development of next‐generation sequencing techniques brought about a new era in killer whale research.

Morin et al. (2010, 2015) sequenced and compared mitochondrial genomes of a global dataset of killer whales in one of the first examples of parallel sequencing using pools of individually indexed DNA libraries. While genome‐wide data from reduced representation methods such as RAD‐seq have been used, the resulting markers are only ever population‐specific and not necessarily representative of diversity across the species' global range (Moura et al. 2014; Reeves et al. 2022). Foote et al. (2016) used whole genome sequences to examine in more detail the genetic differentiation between and within Antarctic and Pacific ecotypes. Importantly, the public archiving of genomic data on the NCBI database after publication has allowed subsequent studies to incorporate previously published genomes and mitogenomes into their datasets (e.g., Filatova et al. 2018; Foote et al. 2019, 2023; Morin et al. 2015; Reeves et al. 2023). Thus, datasets become cumulative over time, allowing new studies to build on data from past work, opening the door to comprehensive global analyses of killer whale genetic diversity and structure.

Despite rapid technological advances, the cost of whole‐genome sequencing still constrains the number of individuals that can be included in population‐scale studies. Targeted SNP genotyping provides a cost‐effective alternative to whole‐genome sequencing while producing data that are readily comparable across laboratories and studies (Morin et al. 2004, 2009). Yet, biases may originate from non‐random sampling of individuals and SNP discovery as well as from biochemical sources during enrichment (Kumar et al. 2012). Since genetic diversity is not evenly distributed across populations, the specific populations in which the SNPs are identified will contribute to SNP ascertainment bias. Consequently, inaccurate results can arise when genotyping other populations for the same loci, particularly when SNPs are initially identified in genetically less diverse populations that have undergone bottlenecks and genetic drift (Lachance and Tishkoff 2013). Therefore, the applicability of any given SNP‐typing method to various, potentially global, population datasets is contingent upon the diversity it encompasses. Furthermore, during enrichment, allelic biases can introduce distortions into the outcomes of population genetic analyses. Certain SNPs and alleles at heterozygous sites are captured more effectively than others due to biochemical and thermodynamic factors such as binding affinity which can lead to an overestimated similarity between samples and, hence, distort insights into populations' evolutionary history (Davidson et al. 2023).

In this note we present the findings of an investigation of allelic and SNP bias in a custom‐designed enrichment capture using genome‐wide biotinylated RNA baits (see Enk et al. 2014) manufactured by myBaits Daicel Arbor Biosciences (Design ID: D10110Orca, Reference number: 210810‐901). The SNPs that the enrichment kit targets were discovered through a RAD‐seq experiment that included samples from the North Pacific resident, Biggs (formerly transient) and offshore ecotypes, Iceland and Marion Island populations (Moura et al. 2014). These SNPs were filtered for linkage disequilibrium by removing SNPs physically separated by < 100 kb on autosomal scaffolds, and removing SNPs in known repetitive and low mappability regions of the killer whale (Oorc_1.1; NCBI Bioproject PRJNA167475; Foote et al. 2015), resulting in a dataset of 1346 unlinked genome‐wide SNPs (Foote and Morin 2016). DNA libraries enriched using the kit have been tested for reliability in estimating pairwise relatedness using a known pedigree of captive Icelandic killer whales (Jourdain et al. 2024) and used for estimating relatedness and structure among Norwegian killer whales (Jourdain et al. 2024) and an extended data set of North Atlantic killer whales (Baumgartner et al. 2025). However, whether enrichment using this kit suffers from allelic bias or SNP ascertainment bias that would reduce its usefulness in some populations or global comparisons is currently unknown.

To assess how the baits captured species‐wide genetic variation we analyzed 25 whole genome sequences (> 10×), each representing a different population across the species' range (see Foote et al. 2019, 2021). Sequences were trimmed with AdapterRemoval v2.3.2 to remove residual adapters and low‐quality regions, retaining reads ≥ 70 bp (Schubert et al. 2016). Cleaned reads were mapped to the high‐quality Norwegian killer whale genome chromosomal assembly (Foote et al. 2022) using BWA‐MEM v0.7.17 (Li and Durbin 2009). Duplicate and ambiguous reads were removed with SAMtools v1.14 (Li et al. 2009). Repeat regions were identified with RepeatMasker v4.1 (Smit et al. 2004) and masked using BEDtools v2.3.0 (Quinlan 2014). Separate files were produced for autosomes and sex chromosomes.

We used ANGSD (Korneliussen et al. 2014) to estimate posterior genotype likelihoods based on allele frequency priors on the autosomes, inferring major and minor alleles. We retained SNPs at polymorphic sites with likelihood ratio test *p*‐values < 1 × 10^−6^ and a minimum minor allele frequency of 0.05. Reads with mapping quality < 30, low base quality (*q* < 30), non‐unique mappings, or flagged as bad (≥ 256) were removed. We also adjusted q‐scores around indels and set a minimum quality score of 50 for excessive mismatches. Genetic covariance matrices were estimated using PCAngsd v1.36.3 (Meisner and Albrechtsen 2018) for both the WGS dataset (obtained using 225,281 unlinked SNPs from the shotgun sequenced genome assemblies) and the targeted baits panel (genomes subsampled 1346 SNPs targeted by the baits).

**FIGURE 1:**
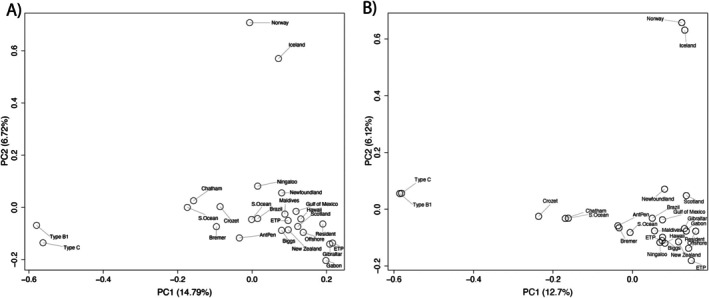
Genetic variation along the first two principal components of a PCA derived from (A) 1346 bait‐targeted SNPs, (B) 225,281 unlinked SNPs from shotgun sequenced genome assemblies (adjusted from figure S5 of Foote et al. 2019). The following abbreviations are used: AntPen, Antarctic Peninsula; ETP, Eastern Tropical Pacific; S.Ocean, Southern Ocean.

We performed principal component analysis (PCA) in R v4.2.1 (RCore Team 2022) to identify clusters based on allele frequency covariance. The PCA based on the targeted baits panel recapitulates the pattern obtained using the WGS dataset of the same samples, though minor variation was observed in samples not included in the original capture protocol (Figure 1A,B, respectively). The proportion of total variance explained by the first two principal components was higher in the targeted baits panel than in the WGS dataset, consistent with the expectation that leading eigenvalues are inflated when fewer markers are used relative to the number of samples (Patterson et al. 2006).

To quantitatively assess structural similarity and test for marker‐specific ascertainment bias in the targeted baits panel, we conducted a Procrustes analysis. We used the protest function in the R package *vegan* (Oksanen et al. 2026) to rotate, scale, and superimpose the PCA coordinates of the targeted baits panel onto those of the WGS dataset. To determine whether observed deviations in genetic clustering were driven by the specific targeted SNPs or simply the reduced number of markers, we generated an empirical null distribution. We randomly subsampled 1346 SNPs from the WGS dataset 100 times, generated a covariance matrix for each subset, and performed a Procrustes test against the WGS PCA to calculate an empirical *p*‐value. Procrustes analysis revealed a high structural correlation between the WGS PCA and the targeted baits panel PCA (*r* = 0.961). However, this correlation fell outside the empirical null distribution of 100 randomly sampled 1346‐SNP panels (range: *r* = 0.966–0.995, empirical *p* < 0.01). This indicates that while the baits captured most distinct genetic variation across the species' range, some variation in excluded samples used in the design of the capture baits remains unrepresented, thereby reducing the true extent of variation captured in the whole genome dataset, especially in lower latitude populations such as off Ningaloo (northwestern Australia).

We conclude that the D10110Orca myBaits kit is a reliable and cost‐effective tool for broad‐scale killer whale population genetics. However, the kit exhibits some bias, as it fails to capture genetic variation in populations not included in the design of the target enrichment capture baits. This bias does not appear to distort major population inferences, as the kit's 1346 SNPs successfully replicated the broad‐scale structure derived from whole genome resequence data. The tool is therefore effective for assessing broad patterns of genetic variation, but may be unsuitable for fine‐scale analyses in divergent, unrepresented populations.

## Author Contributions

**C. D. Baumgartner:** funding acquisition (supporting), investigation (equal), project administration (equal), validation (equal), writing – original draft (equal), writing – review and editing (equal). **O. A. Filatova:** funding acquisition (supporting), validation (equal), writing – original draft (equal), writing – review and editing (equal). **A. D. Foote:** conceptualization (lead), data curation (lead), formal analysis (supporting), funding acquisition (lead), investigation (equal), methodology (equal), project administration (equal), resources (lead), supervision (equal), validation (equal), writing – original draft (equal), writing – review and editing (equal). **E. Jourdain:** validation (equal), writing – review and editing (equal). **F. I. P. Samarra:** validation (equal), writing – review and editing (equal). **I. M. Reeves:** formal analysis (lead), funding acquisition (supporting), investigation (equal), methodology (equal), project administration (equal), resources (supporting), supervision (equal), validation (equal), visualization (lead), writing – original draft (equal), writing – review and editing (equal).

## Funding

A.D.F. was funded by an ERC COG 101045346. C.D.B. was funded by a PhD scholarship from ETH Zurich. This study resulted from a workshop funded by a Laura Corrigan Conservation Grant.

## Conflicts of Interest

The authors declare no conflicts of interest.

## Data Availability

The custom‐designed baits are available from myBaits Daicel Arbor Biosciences using the following codes, design ID: D10110Orca, reference number: 210810‐901. The complete sequencing datasets have been deposited in the National Center for Biotechnology Information (NCBI) or the European Nucleotide Archive (ENA). Please refer to table S1 in Foote et al. (2019) for the specific accession numbers.
